# Development and validation of a nomogram model for predicting the risk of failed manual reduction in distal radius fractures

**DOI:** 10.3389/fsurg.2026.1839273

**Published:** 2026-06-11

**Authors:** Mingyue Fan, Binbin Gu

**Affiliations:** Fuyang Cancer Hospital, Fuyang, China

**Keywords:** AO classification, distal radius fracture, manual reduction, nomogram, risk factors

## Abstract

**Purpose:**

To develop a nomogram model for predicting the risk of failed manual reduction in patients with distal radius fractures (DRF) and to validate its predictive performance.

**Methods:**

A total of 822 patients with DRF were retrospectively reviewed and divided into two groups based on the success or failure of manual reduction. Univariate and multivariate logistic regression analyses were performed to identify independent risk factors. Variables with *P* < 0.05 in multivariate analysis were used to construct a nomogram using the “rms” package in R software. Internal validation was conducted using bootstrap resampling with 1,000 iterations.

**Results:**

Eight independent risk factors were incorporated into the predictive model. The receiver operating characteristic curve showed an area under the curve (AUC) of 0.878 (95% CI: 0.855–0.901), with a sensitivity of 75.4%, a specificity of 82.5%, and a Youden's index of 0.579. The calibration curve demonstrated good agreement between predicted and observed probabilities, with a mean absolute error of 0.005 after bootstrap internal validation (1,000 iterations).

**Conclusion:**

The independent risk factors for failure of manual reduction were determined to be AO classification, severity of swelling, etiology of the fracture, time from injury to reduction, pre-reduction palmar tilt angle, radial shortening height, pre-reduction ulnar deviation angle, and a history of alcohol consumption. The nomogram model constructed based on these factors demonstrates high predictive accuracy for the risk of failed manual reduction in DRF. It has the potential to assist clinicians in more accurately assessing individual patient risk, thereby providing valuable guidance for personalized treatment strategies and clinical decision-making.

## Introduction

Distal radius fracture (DRF) is one of the most common types of fractures encountered in clinical practice. It is highly prevalent among both individuals under 18 and those over 50 years of age. Furthermore, its incidence increases progressively with advancing age in middle-aged and elderly populations (1). Typical clinical manifestations include wrist pain, swelling, deformity, subcutaneous ecchymosis, and limited mobility (2). For common closed DRFs, the primary treatment options consist of manual reduction with plaster immobilization and open reduction internal fixation (3). Most patients achieve satisfactory outcomes following conservative treatment. Conservative management offers notable advantages in terms of safety and cost-effectiveness (4–6); however, a subset of patients ultimately requires surgical intervention. Therefore, identifying the factors contributing to failure of manual reduction is of critical importance. The nomogram typically consists of a series of lines, each representing the influence of a predictive variable. These lines allow for a visual assessment of the impact of different variables on the outcome. Although several studies have investigated predictors of instability or redisplacement after conservative treatment of DRFs (7–11), to our knowledge, no study has developed a nomogram specifically for predicting the failure of manual reduction in the Chinese population. Previous work has identified various risk factors for secondary displacement, including initial displacement, fracture comminution, and bone quality (12–14), but a comprehensive predictive tool integrating multiple variables remains lacking. The present study aims to address this gap by providing a granular analysis of local clinical variables. This model is expected to assist clinicians in more accurately assessing individual patient risk, thereby providing a valuable evidence-based reference for formulating personalized treatment and management plans.

## Materials and methods

### Study design and ethics

A retrospective study was conducted on patients with distal radius fractures who were diagnosed and treated at our hospital from June 2022 to June 2025. This study was conducted in accordance with the Declaration of Helsinki and was approved by the Medical Ethics Committee of Fuyang Cancer Hospital (Approval No. 2025FYSZLYY-IRB-58).

### Inclusion and exclusion criteria

#### Inclusion criteria

1. Diagnosis of a closed distal radius fracture (DRF);
2. All patients first underwent manual reduction, followed by digital radiography (DR) imaging. Surgery was performed if the operative criteria were satisfied;
3. Provision of informed consent by the patient and/or their family members;
4. Availability of complete demographic and follow-up data.

#### Exclusion criteria

1. Open DRF;
2. Presence of combined neurovascular injuries or contraindications to manual reduction;
3. History of previous wrist fracture or surgery;
4. Refusal of treatment or follow-up.

### Definition of manual reduction failure

Manual reduction failure was defined according to three criteria: (1) immediate failure—unacceptable alignment immediately after closed reduction on post-reduction radiographs (i.e., dorsal angulation >10°, radial shortening >3 mm, or intra-articular step-off >2 mm); (2) secondary displacement—loss of acceptable alignment during follow-up (within 2 weeks post-reduction) requiring re-intervention; and (3) conversion to surgery—any case where the treating surgeon decided to proceed to open reduction internal fixation (ORIF) based on the above criteria (15, 16). All patients received standardized cast immobilization (below-elbow cast with neutral wrist position) and follow-up at 1, 2, and 6 weeks. The same immobilization and follow-up protocol was applied to all patients.

### Data collection

Data were extracted from the electronic medical record system. The following variables were collected: sex, age, BMI, injured side, AO classification, severity of limb swelling, cause of fracture, time from injury to reduction, pre-reduction palmar tilt angle, radial shortening height, pre-reduction ulnar deviation angle, presence of osteoporosis, smoking history, alcohol consumption history, hypertension, diabetes, coronary heart disease, arrhythmia, and use of non-steroidal anti-inflammatory drugs (NSAIDs) or glucocorticoids. Swelling severity was graded retrospectively from clinical notes using a standardized three-point scale (mild, moderate, severe) based on the presence of skin wrinkling, palpable bony landmarks, and circumference difference. This grading system has been previously used in similar studies (17). Osteoporosis was defined by a prior DXA T-score ≤ −2.5, a formal diagnosis in the medical record, or current treatment with anti-osteoporotic medications (18). For patients without documented DXA results, the absence of any recorded diagnosis or treatment was classified as “no osteoporosis”, which is acknowledged as a limitation.

### Statistical analysis

Statistical analyses were performed using R software (version 4.3.0). Measurement data conforming to a normal distribution are presented as mean ± standard deviation ($\bar{x}\pm s$), while non-normally distributed measurement data are presented as median (interquartile range, IQR). Normality was assessed using the Shapiro–Wilk test. Categorical data are described as frequency and percentage (%). Intergroup comparisons of categorical data were conducted using the chi-square test. For measurement data, Student's *t*-test or the Mann-Whitney *U*-test was applied for intergroup comparisons based on distribution. For multivariable analysis, all variables with *P* < 0.10 in univariate analysis were entered into a binary logistic regression model using a forward stepwise (likelihood ratio) selection method. The significance level for retention was *P* < 0.05. A nomogram-based risk prediction model for manual reduction failure in DRF was constructed using the “rms” package in R. The predictive performance of the model was assessed using the receiver operating characteristic (ROC) curve. Calibration curves were plotted to evaluate agreement between predicted probabilities and observed outcomes. Decision curve analysis (DCA) was employed to evaluate the net benefit of the model. The significance level (α) was set at 0.05.

## Results

### Patient characteristics

A total of 822 patients were included. The median age was 66.0 years [interquartile range (IQR), 33.0–75.0 years]. There were 312 male patients (37.96%) and 510 female patients (62.04%). Among these, 480 (58.39%) were classified into the failure of manual reduction group and 342 (41.61%) into the success group.

### Univariate analysis

Univariate analysis revealed statistically significant differences (*P* < 0.05) between the failure and success groups in the following variables: age, alcohol consumption, AO classification, severity of limb swelling, cause of fracture, time from injury to reduction, pre-reduction palmar tilt angle, radial shortening height, pre-reduction ulnar deviation angle, use of NSAIDs, hypertension, coronary heart disease, and diabetes. Results for osteoporosis, sex, and other variables are shown in Table 1. Details are presented in Table 1.

**Table 1:** Results of the univariate analysis for failed manual reduction in distal radius fractures.

Variables	Total quantity	Success group	Failure group	*χ2*	*P*
Sex (%)				2.046	0.175
Male	510 (62.0)	288 (60.0)	222 (64.9)		
Female	312 (38.0)	192 (40.0)	120 (35.1)		
Age [median (IQR)]	66.0 [33.0, 75.0]	61.0 [31.0, 74.0]	68.0 [38.0, 76.0]	−2.872*	0.004
BMI [median (IQR)]	25.6 [22.3, 27.5]	25.6 [22.3, 27.5]	25.6 [22.3, 27.5]	0.516*	0.516
Side of injury (%)				1.338	0.279
Left	305 (37.1)	186 (38.8)	119 (34.8)		
Right	517 (62.9)	294 (61.3)	223 (65.2)		
Osteoporosis (%)				0.992	0.355
No	471 (57.3)	282 (58.8)	189 (55.3)		
Yes	351 (42.7)	198 (41.2)	153 (44.7)		
Smoking (%)				3.173	0.088
No	543 (66.1)	329 (68.5)	214 (62.6)		
Yes	279 (33.9)	151 (31.5)	128 (37.4)		
Alcohol drinking (%)				12.607	0.001
No	588 (71.5)	366 (76.2)	222 (64.9)		
Yes	234 (28.5)	114 (23.8)	120 (35.1)		
AO classification (%)				141.207	<0.001
A	139 (16.9)	116 (24.2)	23 (6.7)		
B	237 (28.8)	187 (39.0)	50 (14.6)		
C	446 (54.3)	177 (36.9)	269 (78.7)		
Swelling (%)				94.528	<0.001
Mild	126 (15.3)	103 (21.5)	23 (6.7)		
Moderate	231 (28.1)	173 (36.0)	58 (17.0)		
Severe	465 (56.6)	204 (42.5)	261 (76.3)		
Precipitating factor (%)				34.566	<0.001
Fall from standing height	471 (57.3)	239 (49.8)	232 (67.8)		
Motor vehicle accident	123 (15.0)	72 (15.0)	51 (14.9)		
Fall from height	228 (27.7)	169 (35.2)	59 (17.3)		
Time from injury to reduction [median (IQR)]	2.5 [1.9, 3.2]	2.3 [1.8, 3.0]	2.6 [2.0, 3.5]	−4.688*	<0.001
Pre-reduction volar tilt [median (IQR)]	−5.0 [−8.0, 6.0]	−4.0 [−7.0, 8.0]	−6.0 [−8.0, 5.0]	−4.218*	<0.001
Radial height loss [median (IQR)]	6.0 [4.0, 7.0]	6.0 [4.0, 7.0]	6.0 [5.0, 8.0]	−5.941*	<0.001
Pre-reduction ulnar inclination [median (IQR)]	12.0 [9.0, 15.0]	14.0 [11.0, 18.0]	10.0 [8.0, 13.0]	−13.249*	<0.001
Glucocorticoids (%)				0.962	0.416
No	777 (95.2)	460 (95.8)	317 (94.3)		
Yes	39 (4.8)	20 (4.2)	19 (5.7)		
NSAIDs (%)				29.545	<0.001
No	584 (71.6)	378 (78.8)	206 (61.3)		
Yes	232 (28.4)	102 (21.2)	130 (38.7)		
Hypertension (%)				28.05	<0.001
No	447 (54.8)	300 (62.5)	147 (43.8)		
Yes	369 (45.2)	180 (37.5)	189 (56.2)		
Coronary artery disease (%)				10.57	0.002
No	625 (76.6)	387 (80.6)	238 (70.8)		
Yes	191 (23.4)	93 (19.4)	98 (29.2)		
Arrhythmia (%)				2.267	0.162
No	714 (87.5)	427 (89.0)	287 (85.4)		
Yes	102 (12.5)	53 (11.0)	49 (14.6)		
Diabetes mellitus (%)				19.896	<0.001
No	543 (66.5)	349 (72.7)	194 (57.7)		
Yes	273 (33.5)	131 (27.3)	142 (42.3)		

The asterisk (*) indicates the Zc value.

### Multivariate analysis and independent risk factors

The variables with *P* < 0.10 in univariate analysis were included as independent variables in a binary logistic regression model, with the success or failure of manual reduction serving as the dependent variable. The variable assignment is detailed in Table 2. The results indicated that a history of alcohol consumption, AO classification, severity of limb swelling, cause of fracture, time from injury to reduction, pre-reduction palmar tilt angle, radial shortening height, and pre-reduction ulnar deviation angle were all independent influencing factors for failure of manual reduction (*P* < 0.05). The strongest predictor in the nomogram was AO type C (point score: 100), followed by severe swelling (point score: 85). Details are presented in Table 3.

**Table 2:** Assignment of variables in the multivariate logistic regression analysis.

Variable	Assignment situation
Outcome Variable	Success = 0,Failure = 1
Alcohol drinking	Yes = 1,No = 0
AO classification	A = 1,B = 2,C = 3
Swelling	Mild = 1,Moderate = 2,Severe = 3
Precipitating factor	Fall from standing height = 1,Motor vehicle accident = 2,Fall from height = 3
Hypertension	Yes = 1,No = 0
Coronary artery disease	Yes = 1.No = 0
Diabetes mellitus	Yes = 1,No = 0

**Table 3:** Results of the multivariate logistic regression analysis.

Variable	B	SE	Waldχ2	OR	Lower limit of 95%CI	Upper limit of 95%CI	*P*-value
Constant	−3.649	0.712	26.264	0.026	0.006	0.103	0.000
Age	0.004	0.005	0.765	1.004	0.995	1.013	0.382
Alcohol drinking	0.760	0.216	12.446	2.139	1.407	3.278	0.000
AO classification	1.059	0.149	50.375	2.885	2.166	3.892	0.000
Swelling	0.684	0.144	22.680	1.982	1.502	2.641	0.000
Precipitating factor	−0.339	0.114	8.900	0.712	0.569	0.889	0.003
Time from injury to reduction	0.368	0.114	10.457	1.446	1.158	1.811	0.001
Pre-reduction volar tilt	−0.025	0.012	4.324	0.976	0.953	0.998	0.038
Radial height loss	0.179	0.040	20.007	1.196	1.107	1.296	0.000
Pre-reduction ulnar inclination	−0.268	0.027	101.117	0.765	0.724	0.804	0.000
NSAIDs	0.449	0.318	1.988	1.566	0.841	2.933	0.159
Hypertension	0.271	0.291	0.870	1.311	0.741	2.321	0.351
Coronary artery disease	−0.446	0.320	1.945	0.640	0.340	1.194	0.163
Diabetes mellitus	*0* *.* *466*	0.256	3.309	1.593	0.965	2.637	0.069

### Nomogram construction and performance

**Figure 1:**
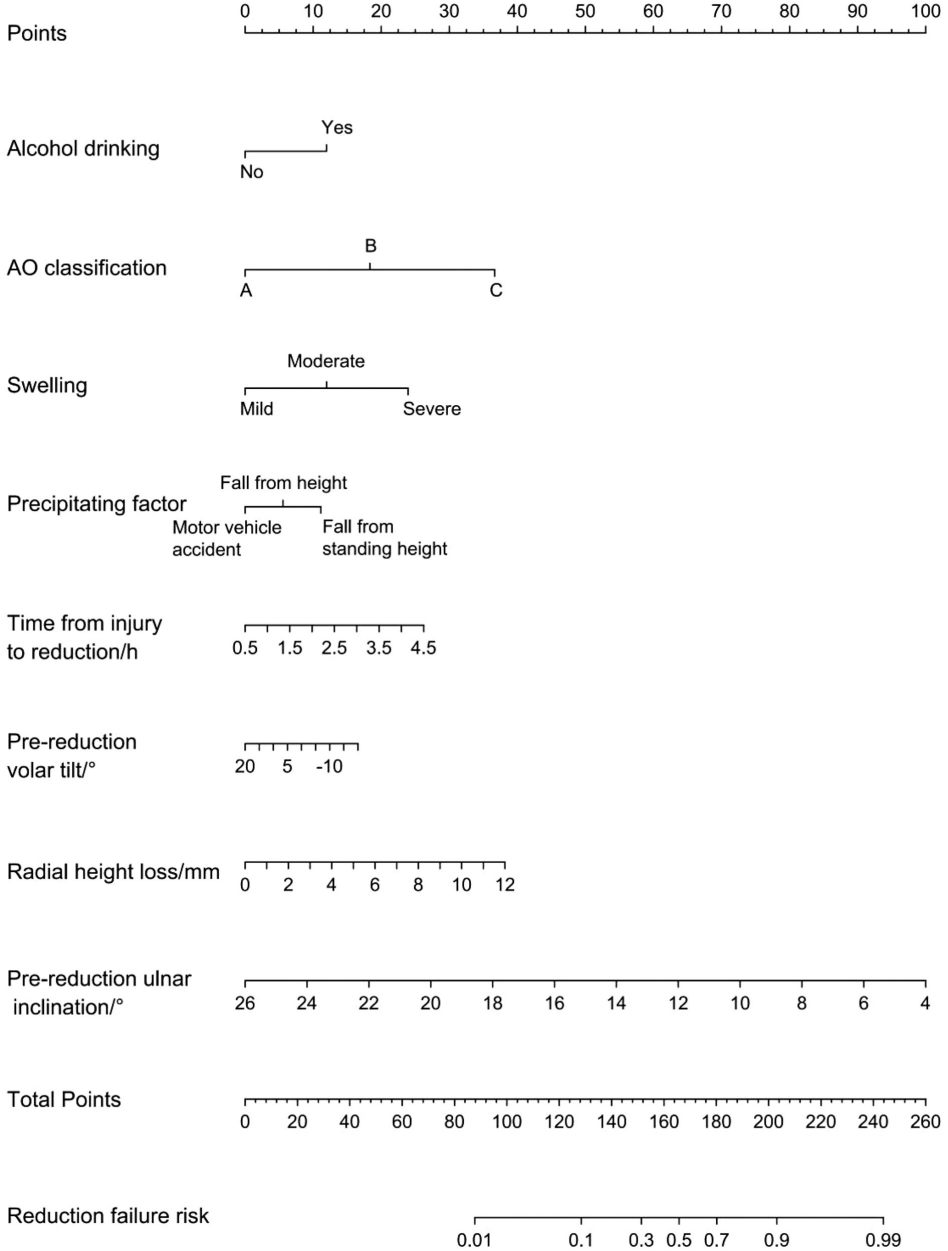
Nomogram for predicting the risk of manual reduction failure in distal radius fractures.

**Figure 2:**
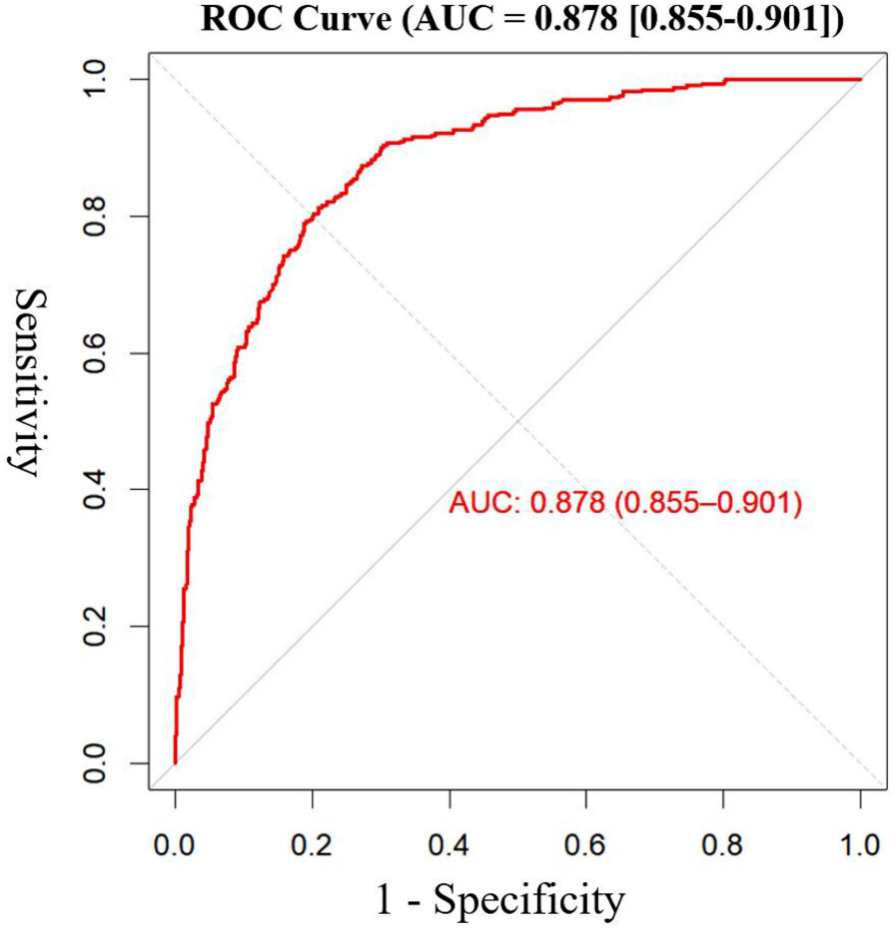
Receiver operating characteristic (ROC) curve.

**Figure 3:**
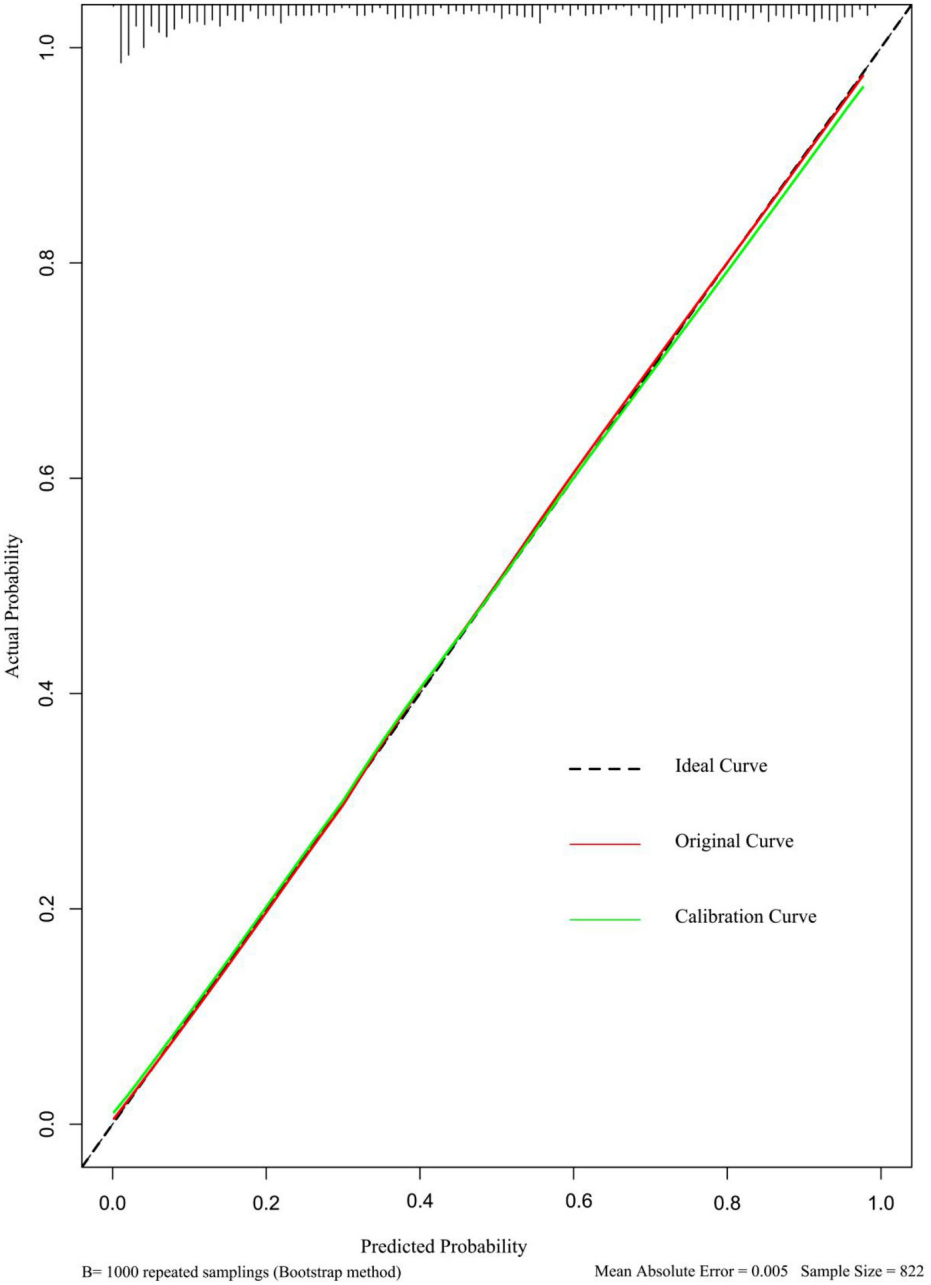
Calibration curve.

**Figure 4:**
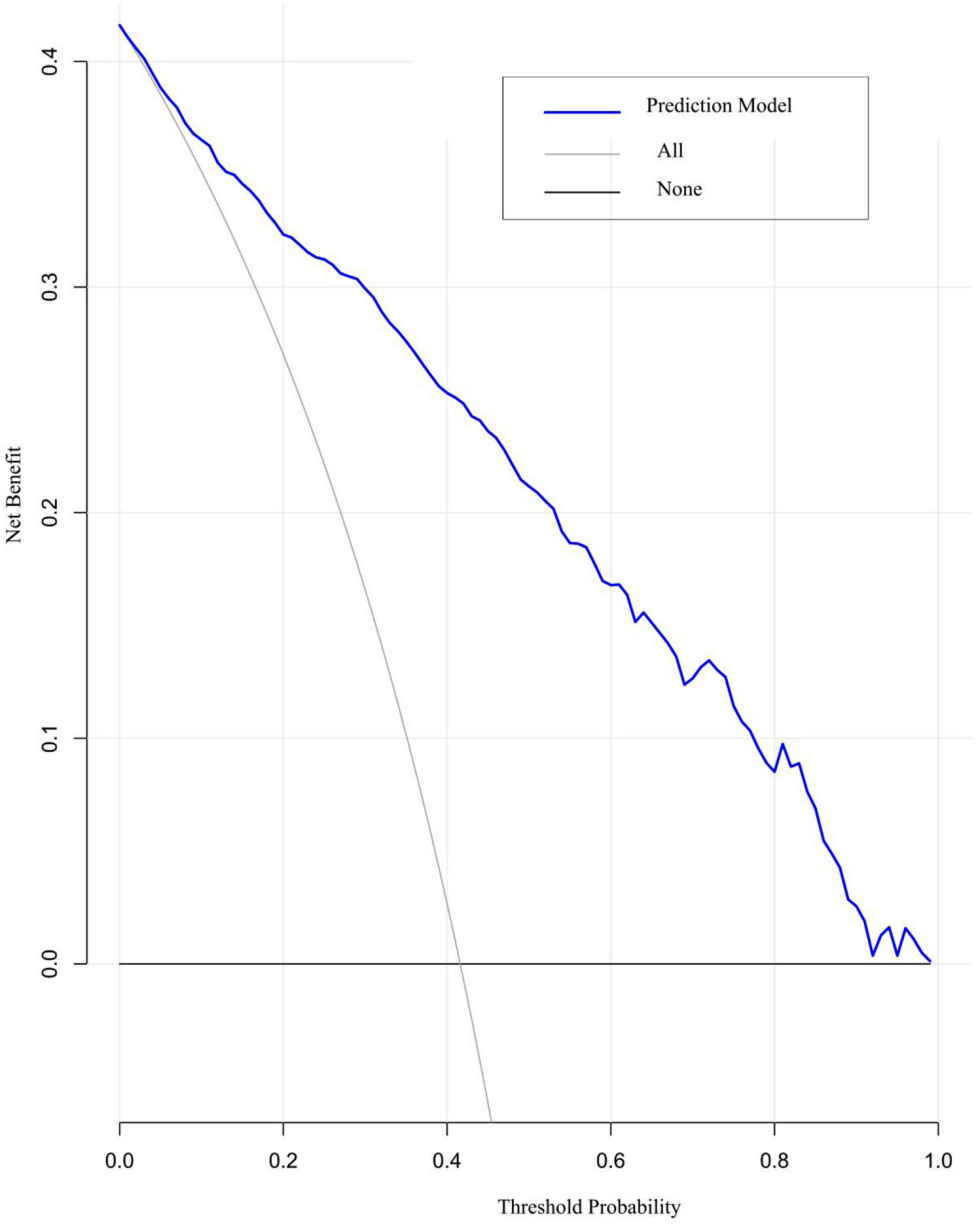
Decision curve analysis (DCA).

Based on the binary logistic regression model, variables with statistical significance were selected as predictors to construct a nomogram-based risk prediction model (Figure 1). The model was validated using a ROC curve. The developed nomogram demonstrated good predictive accuracy for failure of manual reduction (Figure 2), with an AUC of 0.878 (95% CI: 0.855–0.901). The sensitivity and specificity were 75.4% and 82.5%, respectively, yielding a Youden's index of 0.579. The optimal cutoff value of the nomogram total point score was 156, at which the sensitivity was 78.2% and specificity was 80.1%. Patients with a score ≥156 are recommended for primary open reduction internal fixation (ORIF) without an attempt at closed reduction. Furthermore, the calibration curve for the nomogram, assessed via internal validation using bootstrap resampling with 1,000 iterations, showed a mean absolute error of 0.005 (Figures 3, 4). Bootstrap-corrected AUC was 0.872 (95% CI: 0.848–0.896), indicating robust internal validity. These results indicate that the nomogram possesses favorable predictive performance and calibration. The following clinically important variables were not included in the final model because they were not routinely recorded in our retrospective dataset: intra-articular step-off (mm), ulnar variance, volar cortex restoration, and associated ulnar styloid fracture.

## Discussion

Conservative treatment is the preferred initial approach for closed distal radius fractures. It is generally indicated for non-displaced or minimally displaced extra-articular fractures, some stable intra-articular fractures with larger fragments, and elderly patients with lower functional demands for wrist mobility (19). During manual reduction, the patient is placed in a supine or seated position. Traction is applied to both sides of the fracture site to disimpact the fragments. Reduction is then achieved through techniques such as lifting/pressing, nudging displaced fragments, or correcting angulation (20). Following reduction, immobilization is typically achieved using a plaster cast or splint to maintain stability of the fracture. For patients in whom manual reduction fails or where an acceptable reduction cannot be achieved and maintained by closed means alone, surgical intervention becomes necessary (21). The primary goals of surgery are to restore anatomical alignment and articular surface congruity. Stability is then maintained using internal or external fixation devices to promote fracture healing and functional recovery of the wrist. While surgical management offers significant advantages in certain cases, it is not without drawbacks, as postoperative complications can occur and may pose additional risks to the patient. Conservative management, compared to surgery, offers advantages such as simplicity, cost-effectiveness, and often a quicker initial recovery (22, 23). Therefore, in the treatment of closed distal radius fractures, the specific fracture characteristics and clinical requirements should be comprehensively evaluated. An attempt at manual reduction is often warranted. Surgical intervention should be considered if secondary displacement occurs or if the post-reduction alignment fails to meet acceptable functional criteria.To better synthesize the findings, we grouped the identified risk factors into three categories: (1) Patient factors: history of alcohol consumption, osteoporosis (although not retained in the final model), and comorbidities; (2) Initial injury dynamics: AO classification, cause of fracture, and time from injury to reduction; (3) Post-reduction radiographic parameters: pre-reduction palmar tilt angle, radial shortening height, and pre-reduction ulnar deviation angle based on previous classification frameworks (8).Among all predictors, AO classification (specifically type C) emerged as the strongest predictor in the nomogram, contributing the highest point value. This reflects the inherent instability of comminuted intra-articular fractures, which often require surgical stabilization regardless of reduction attempts, consistent with prior reports (24).Regarding the variable “time from injury to reduction”, clinically, delayed reduction allows the fracture hematoma to organize and form early fibrous adhesions, which reduce fragment mobility and make disimpaction difficult (25). Therefore, for patients presenting with prolonged pre-reduction intervals (e.g., >3 h, which is above the median of 2.5 h in our cohort) along with other risk factors, clinicians should consider a lower threshold for primary open reduction internal fixation (ORIF).The findings of this present study indicate that a history of alcohol consumption, AO classification, severity of limb swelling, cause of the fracture, time from injury to reduction, pre-reduction palmar tilt angle, radial shortening height, and pre-reduction ulnar deviation angle are all influential factors contributing to failure of manual reduction. Numerous prior studies have demonstrated that alcohol can disrupt the balance between osteoclastic bone resorption and osteoblastic bone formation, leading to reduced bone density, destruction of trabeculae, osteoporosis, increased bone fragility, and a heightened risk of comminuted fractures, thereby increasing the difficulty of manual reduction (18, 26). In long-term heavy drinkers, clinical manifestations primarily include decreased muscle tone, muscle atrophy, and weakness (27). Such patients may be unable to provide the basic postural maintenance force required during reduction, and the stabilizing “dynamic splinting” effect of muscles on fracture fragments is also lacking post-reduction. Furthermore, alcohol consumption leads to reduced reaction capacity, consequently increasing the incidence of hazardous events such as traffic accidents and falls (28). The result of this study, identifying a history of alcohol consumption as a risk factor for failure of manual reduction in DRF, may be associated with the aforementioned reasons. Severe swelling indicates significant local soft tissue injury and marked hematoma formation. Research by Horoz et al. (17) found that swelling elevates compartment pressure, creating an encapsulating and compressive effect on fracture fragments. While this circumferential pressure may offer some temporary stabilization, it also acts like a continuously tightening “sac,” trapping fragments within the edematous soft tissue and making it difficult to “manipulate” them back into position externally. Simultaneously, swelling and pain lead to muscle spasm and rigidity. Stiffened muscle groups lose elasticity and cannot be effectively lengthened during traction, thereby failing to provide sufficient space for disengaging overlapping or impacted fracture ends (29). Additionally, in patients with severe preoperative limb swelling, successful manual reduction may be followed by a decrease in swelling, causing the cast to become relatively loose. If the cast is not replaced promptly in some patients, secondary displacement of the fracture can occur, increasing the difficulty of maintaining reduction. Such patients often require surgical intervention. The AO classification directly reflects fracture complexity and stability. Type C fractures are complete intra-articular fractures, characterized primarily by complete disruption and comminution of the radiocarpal articular surface (30). The mechanisms leading to reduction difficulty and high failure rates include: comminuted articular fragments lose the integral support of the subchondral bone, resulting in multiple free pieces that cannot be precisely aligned through ligamentotaxis; articular comminution often extends into the metaphysis, causing critical comminution of the volar and/or dorsal cortices. The fracture site thus lacks a stable “bony wall” to support the reduced position, making it highly susceptible to recurrent collapse and shortening under axial load; an intact joint capsule and wrist ligaments can aid in reducing and stabilizing articular fragments through the “ligamentotaxis” effect during traction. Type C fractures are frequently associated with avulsion or failure of these key ligaments (e.g., radioscaphocapitate, radiolunotriquetral ligaments) from the bone fragments, rendering this important indirect reduction mechanism ineffective (24). A decreased pre-reduction ulnar deviation angle and increased radial shortening height often indicate loss of radial axial stability (31), alteration of the wrist biomechanical axis making maintained reduction difficult, and possible concomitant triangular fibrocartilage complex (TFCC) injury further compromising distal radioulnar joint stability. Dorsal angulation (increased negative value) of the pre-reduction palmar tilt indicates dorsal displacement of the fracture, frequently accompanied by dorsal cortical comminution and lack of support; laxity or injury of the volar ligaments predisposes to dorsal redisplacement after reduction; and imbalance in wrist flexor and extensor muscle forces concentrates dynamic stress dorsally. These factors collectively increase the difficulty of manual reduction, consistent with the findings of Plant (32) and Costa (33) et al. In the initial stage post-fracture, the hematoma is liquid or semi-gelatinous, providing some “lubrication” that allows fragments to move and be reduced relatively easily under manual traction. As time progresses, the hematoma begins to organize; a fibrin network forms and gradually contracts, “adhering” the fracture fragments to the surrounding soft tissue (25). This early fibrous connection reduces fragment mobility. During manual traction, the force is dispersed throughout the hematoma rather than being effectively transmitted to the fracture fragments themselves, making disimpaction difficult. The “cause of fracture” essentially reflects a combination of injury energy and patient population characteristics. Elderly individuals with osteoporosis often sustain fractures from low-energy trauma such as minor falls. The osteoporotic bone has low strength and high brittleness, often resulting in comminuted fractures (particularly intra-articular). Reduction lacks stable bony support, and even temporarily achieved reduction is highly prone to recurrent collapse and displacement, leading to a high failure rate. This aligns with findings from a study by Jung et al. (34). In contrast, middle-aged and younger adults with relatively better bone quality are more likely to sustain injuries from high-energy trauma like traffic accidents. Although the violence involved is greater, the better bone quality in these patients may result in more “clean” fracture patterns (e.g., simple transverse or wedge fractures) rather than severe comminution. This makes it more feasible to restore alignment and apposition through manual techniques (35). Moreover, high-energy trauma may lead to more complex open fractures or polytrauma, cases which are often directly treated surgically and thus not included in the cohort analyzed for manual reduction. Therefore, the patients ultimately included in the analysis might be those with relatively controlled injuries who were candidates for closed reduction, and their baseline fracture patterns may, in fact, be less complex than the comminuted fractures seen in the elderly.

A nomogram is commonly employed to visualize the results of multivariate regression analysis. It serves as a graphical tool to intuitively demonstrate the degree of influence that multiple variables exert on an outcome (e.g., disease risk, survival rate, probability of an event) (36). Typically consisting of a series of scaled lines, each representing a predictor variable, a nomogram allows for a direct visual assessment of the relative impact of different factors on the outcome. It facilitates a clearer understanding of the weight assigned to each variable, thereby aiding in the interpretation of the multivariate model's findings. In this study, a nomogram prediction model was constructed based on identified risk factors including alcohol consumption history, AO classification, severity of swelling, cause of fracture, time from injury to reduction, pre-reduction palmar tilt angle, radial shortening height, and pre-reduction ulnar deviation angle. The model was subsequently validated. The area under the ROC curve was 0.878 (95% CI: 0.855–0.901), indicating that the model possesses good discriminative ability, calibration, and overall favorable clinical predictive performance.

## Strengths and limitations

This study has several strengths, including a large sample size and the first nomogram for failure of manual reduction in DRFs. However, limitations should be acknowledged. First, as a retrospective study, the range of variables included may not be exhaustive. Specifically, we could not retrieve data on intra-articular step-off, ulnar variance, volar cortex restoration, or associated ulnar styloid fractures due to incomplete radiographic documentation. These parameters are clinically important for DRF stability, and their absence may limit the model's comprehensiveness. Future prospective studies should incorporate these variables. Second, the investigation was conducted at a single center, and although internal bootstrap validation was performed, external validation is lacking. Third, the assessment of swelling severity was based on retrospective chart review, which may introduce subjectivity; a prospective grading protocol would be more reliable. Fourth, osteoporosis was treated as a binary variable because DXA T-scores were not available for all patients, which may have reduced its analytical value. A more stratified approach using T-scores would be preferable. Therefore, future large-scale, multi-center prospective studies are warranted to further validate these findings.

## Conclusion

In summary, factors including a history of alcohol consumption, AO classification, severity of swelling, etiology of the fracture, time from injury to reduction, pre-reduction palmar tilt angle, radial shortening height, and pre-reduction ulnar deviation angle are identified as risk factors for failure of manual reduction. As these parameters are readily obtainable in clinical practice, the nomogram model developed based on these factors demonstrates high clinical applicability. It can provide clinicians with a practical tool for patient assessment and offer valuable guidance for personalized treatment and management strategies.

## Data Availability

The raw data supporting the conclusions of this article will be made available by the authors, without undue reservation.
